# Co-expression Network Analysis Identifies Four Hub Genes Associated With Prognosis in Soft Tissue Sarcoma

**DOI:** 10.3389/fgene.2019.00037

**Published:** 2019-02-04

**Authors:** Zhenhua Zhu, Zheng Jin, Yuyou Deng, Lai Wei, Xiaowei Yuan, Mei Zhang, Dahui Sun

**Affiliations:** ^1^Department of Orthopaedic Trauma, The First Hospital of Jilin University, Changchun, China; ^2^Department of Immunology, College of Basic Medical Sciences, Jilin University, Changchun, China; ^3^Department of Urology, The First Hospital of Jilin University, Changchun, China; ^4^College of Computer and Control Engineering, Nankai University, Tianjin, China; ^5^College of Chemistry, Jilin University, Changchun, China

**Keywords:** soft tissue sarcoma, weighted gene co-expression analysis, RRM2, BUB1B, CENPF, KIF20A

## Abstract

**Background:** Soft tissue sarcomas (STS) are heterogeneous tumors derived from mesenchymal cells that differentiate into soft tissues. The prognosis of patients who present with an STS is influenced by the regulation of a complex gene network.

**Methods:** Weighted gene co-expression network analysis (WGCNA) was performed to identify gene modules associated with STS (Samples = 156).

**Results:** Among the 11 modules identified, the black and blue modules were highly correlated with STS. However, using preservation analysis, the black module demonstrated low preservation, therefore the blue module was chosen as the module of interest. Furthermore, a total of 20 network hub genes were identified in the blue module, 12 of which were also hub nodes in the protein-protein interaction network of the module genes. Following additional verification, 4 of 12 genes (*RRM2, BUB1B, CENPF*, and *KIF20A*) demonstrated poorer overall survival and disease-free survival rate in the test datasets. In addition, gene set enrichment analysis (GSEA) demonstrated that samples with a high level of blue module eigengene (ME) were enriched in cell cycle and metabolism associated signaling pathways.

**Conclusion:** In summary, co-expression network analysis identified four hub genes associated with prognosis for STS, which may diminish the prognosis by influencing cell cycle and metabolism associated signaling pathways.

## Introduction

Soft tissue sarcoma (STS) is a rare group of tumors that accounts for approximately 1% of adult cancers. In 2009, it was estimated that 3,300 new cases were diagnosed in Britain and 10,000 in the United States (Linch et al., 2014). There are approximately 50 STS subtypes, which differ significantly in their disease presentation, response to currently available treatments and risk of tumor progression (Casali et al., 2018). Multiple factors have been reported to be related to the progression of STS, including capillary morphogenesis gene 2 (CMG2) (Greither et al., 2017), HIF-2α protein (Nakazawa et al., 2016), epidermal growth factor receptor (EGFR) protein (Yang et al., 2017) and microRNAs (Smolle et al., 2017). However, no molecular biomarkers have been defined for predicting the prognosis of the disease in clinical. Therefore, a better understanding of the molecular pathogenesis is required.

To date, microarray-based expression data have been used to identify genes related to tumor progression and prognosis. Takahashi et al. (2014) identified 25 survival-associated genes using a knowledge-based filtering and multiple testing approach. Beck et al (2010) has reviewed the manner in which gene expression profiling has been used to understand sarcoma pathobiology and identify clinically useful biomarkers. However, most studies have focused on screening genes that have different patterns of expression with explanations gained from gene ontology (GO) analysis. Such approaches, however, have failed to address the large number of interconnections between genes, because genes with similar expression profiles are most likely to function closely together. Therefore, weighted gene co-expression network analysis (WGCNA) clusters genes co-expressed in a network, based on similarities in expression profiles among samples and in clinical traits, to define sub-network regions (known as modules) (Langfelder and Horvath, 2008).

In this study, we utilized WGCNA to identify the most relevant module in STS. Key genes in the module were identified and validated using survival and protein-protein interaction (PPI) analyses. These key genes may shed new light on the biological mechanisms underlying STS progression and could potentially be used as prognostic biomarkers or therapeutic targets.

## Materials and Methods

### Study Design and Data Collection

**FIGURE 1:**
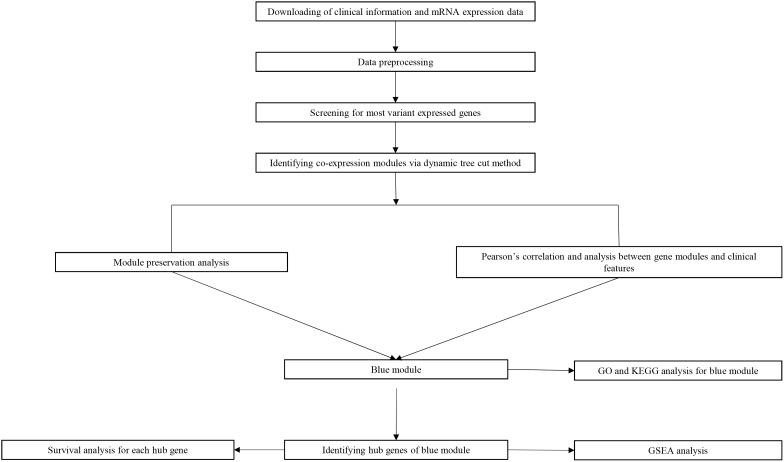
Flow diagram of strategy for data preparation, preprocessing and analysis used in this study.

Study design, data preparation, preprocessing, analysis and validation are described in a flowchart (Figure 1). Core codes used to reproduce the results were provided in Supplementary Table S1. Firstly, normalized RNAseq data and associated clinical data were downloaded from the NCBI Gene Expression Omnibus (GEO). Dataset GSE21122 (Barretina et al., 2010), which was generated using an Affymetrix human genome U133A microarray (HG-U133A), was used as a training set to construct the co-expression network and identify key modules in this study. This dataset included 149 STS samples and 9 normal fat tissue samples. The STS samples contained 116 different types of liposarcoma and 34 malignant fibrous histiocytomas (MFHs). Most STSs (68.8%) were primary tumors at the time of sample procurement from patients whose mean age was 56 years. In addition, two test datasets were used to test the preservation of identified modules and survival significance of hub genes. The first one, which included RNA sequencing data and associated clinical information of 265 STS samples, were downloaded from The Cancer Genome Atlas (TCGA) database^1^. The other one, GSE21050 dataset (Chibon et al., 2010), which included RNA sequencing data and associated clinical information of 310 STS samples were downloaded from the NCBI GEO.

### Data Preprocessing

Firstly, we extracted training expression data from the GSE21122 MINiML file. The expression data was background corrected using the Robust Multi-array Average (RMA) algorithm and log base 2 normalized. The data were then checked to ascertain whether there was a batch effect. No apparent batch effect was observed after analysis of expression clusters, box plots and principal components analysis (PCA) (Supplementary Figure S1). In order to detect outliers for WGCNA analysis, sample network was calculated based on squared Euclidean distance. The connectivity of each sample was defined as the sum of the connectivity of that sample with all other samples. Outliers were identified after normalization of the connectivity of each sample, by use of the threshold z.k < 0.6. Generally, genes whose expression varies greatly are more biologically relevant. To reduce background noise, we selected genes that were varied expressed across samples and removed those whose expression was the same across samples. The median absolute deviation (MAD) was calculated for each gene as a robust measure of variability. Then, genes were sorted based on the MAD value and the top 3,000 ranked genes were used for the subsequent WGCNA analysis.

### Co-expression Network Construction and Module Preservation Analysis

The WGCNA package (Langfelder and Horvath, 2008) was used to construct the co-expression network. The concordance of genes in the expression dataset was measured with Pearson correlation, then the Pearson correlation matrix was transformed to weighted network with the power adjacency function. The first step in this process was selection of an appropriate soft power, in which strong connections between genes are promoted and weak connections penalized, so as to transform the network into one meeting the requirements of a scale-free network. Modules were identified using the dynamic tree-cutting function with a deepSplit argument value of 2 and a minimum size cutoff of 30. To test whether the identified modules were stable in the test TCGA dataset, the downloaded fragments per million (FPKM) expression data of 265 samples were transformed to the transcripts per million (TPM). A total of 2704 common genes in the training and TCGA datasets were used for preservation analysis. The module Preservation function (nPermutations = 200) of the WGCNA package (Langfelder et al., 2011) was utilized, in which the preservation statistic Zsummary was used to quantify the preservation of gene modules between datasets.

### Finding Modules of Interest and Functional Annotation

Because the module eigengene (ME) provides the most appropriate synopsis of gene expression profiles of any given module, we correlated MEs with clinical traits. In this study, clinical traits refer to whether the sample was a STS or normal fat tissue. Correlations were then calculated using linear regression model. The modules for which the eigengenes showed high correlation were chosen as the modules of interest. In an attempt to ascertain possible mechanisms of genes within a module affecting STS progression, functional enrichment analyses using the KEGG and GO databases of the hub module was performed with the “clusterProfile” package in R (Yu et al., 2012).

### Identification of Hub Genes and Correlation Analysis

Hub genes are those that have a high degree of intra-module connectivity. In this study, hub genes were defined as the 20 module genes with highest connectivity in the interested module. A PPI network was constructed in order to identify hub nodes by uploading all genes in the hub module to the Search Tool for the Retrieval of Interacting Gene (STRING) database^2^. The PPI network was then imported into the Cytoscape software platform and a comprehensive analysis of the relationship between nodes was performed using the Maximal Clique Centrality (MCC) function, reported to be the most effective method of finding hub nodes in a co-expression network (Chin et al., 2014), within the “cytoHubba” application. In this way, the most cohesive genes were marked as “first stage nodes.” In the PPI network of blue module genes, the 30 most highly ranked nodes were identified as “first stage nodes.” Genes that were defined as both hub genes in the module and “first stage nodes” in the PPI network were chosen as primary hub genes.

### Survival Analysis and Efficacy Evaluation

The internet tool, Gene Expression Profiling Interactive Analysis (GEPIA)^3^, was used to perform overall survival and disease-free survival analyses for all hub genes. The platform utilizes all expression data and survival information of the TCGA database. Users are able to accomplish survival analysis by simply submitting a gene name and selecting a tumor type. Patients were divided into two groups (high vs. low) based on the hub gene expression level in comparison to the mean expression level of that hub gene. Furthermore, dataset GSE21050, which includes 310 STS samples in which metastasis status and survival time were provided, was used to test the significance of hub genes for metastasis survival. A Kaplan-Meier survival plot was constructed using the “survival” package in R (Li, 2003). Differential expression between STS and normal tissue in the training set was plotted as a box plot graph.

### Gene Set Enrichment Analysis (GSEA)

In the training data set, 156 samples were dichotomized into two groups (High vs. Low) based on the ME value of blue module in comparison to the mean ME level of blue module of all samples. GSEA was then performed between the two groups. The 3,000 most variable genes from the WGCNA were imported for enrichment. In this way, GSEA was used to validate the results of GO and KEGG analysis of the blue module. The cut-off criterion for GSEA was FDR < 0.05.

## Results

### Co-expression Network Construction and Module Preservation Analysis

**FIGURE 2:**
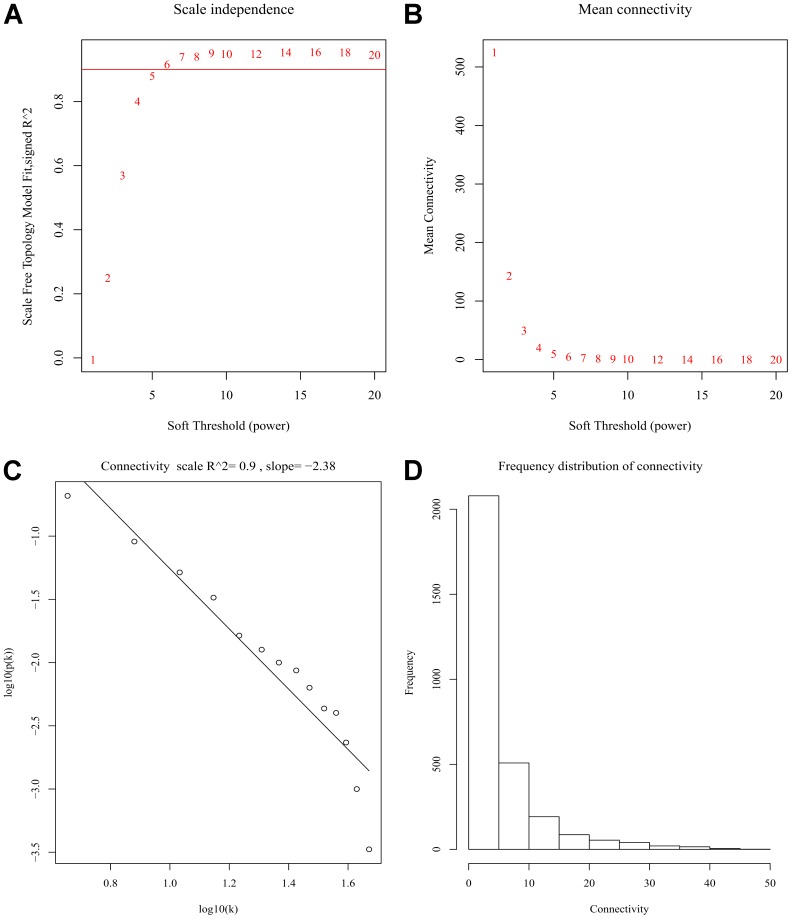
Determination of soft-thresholding power in the weighted gene co-expression network analysis (WGCNA). **(A)** Analysis of scale-free fit index for various soft-thresholding powers (β). **(B)** Analysis of mean connectivity for various soft-thresholding powers. **(C)** Linear model fitting of *R*^2^ index showed good quality of fit. **(D)** Frequency distribution of connectivity.

**FIGURE 3:**
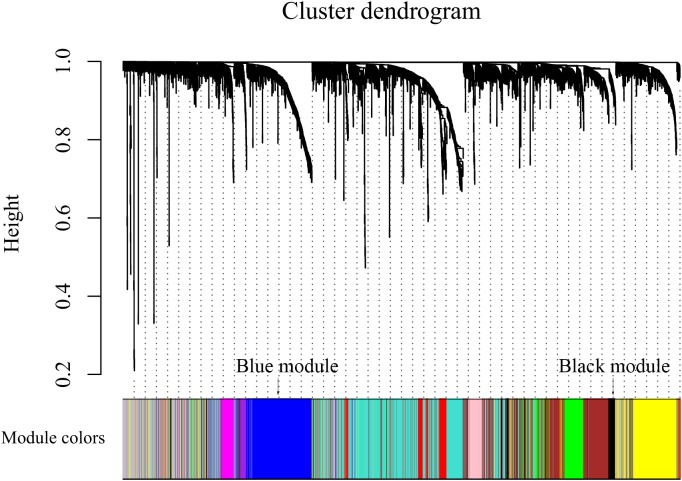
Color coding of co-expression network modules for mRNAs.

After discarding two outlier samples (GSM528297 and GSM528333), WGCNA was performed on the 3,000 most variable genes of 156 samples. Soft threshold power was set to 6, in which *R*^2^ was 0.916, ensured a scale-free network (Figure 2). Following this, 11 co-expression modules were identified, ranging in size from 43 to 669 genes (with each module assigned a color) (Figure 3).

**FIGURE 4:**
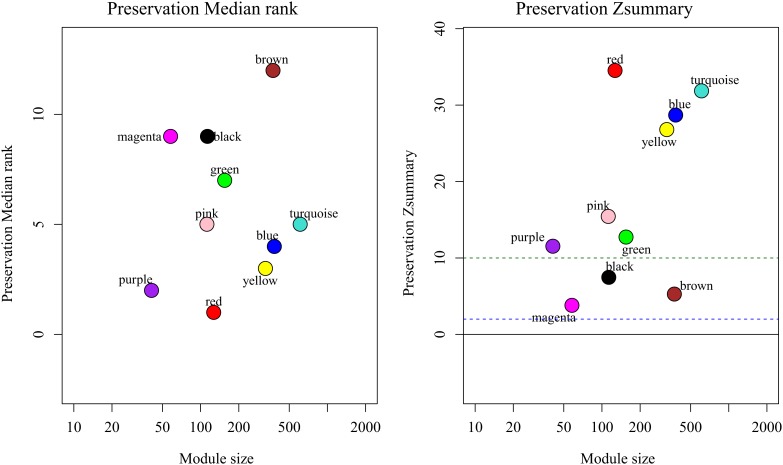
medianRank and Zsummary statistics of the most variant gene modules in module preservation. In the preservation medianRank graph (left), a medianRank value close to zero indicates a high degree of module preservation. In the preservation Zsummary graph (right), the dashed black lines indicate the thresholds *Z* = 2, 10. These horizontal lines indicate Zsummary thresholds for strong evidence of conservation (above 10) and for low to moderate evidence of conservation (above 2).

By comparing the training dataset GSE21122 with the TCGA test dataset, we were able to establish whether the co-expression modules produced in the training dataset could be reproduced in the test dataset through summary preservation statistics. Three modules (black, brown, and magenta) demonstrated poor preservation with each Zsummary statistic < 10. The remaining modules, including the blue module were stable enough, suggesting they were preserved between the training data set and the test data set (Figure 4).

### Finding Modules of Interest and Functional Annotation

**FIGURE 5:**
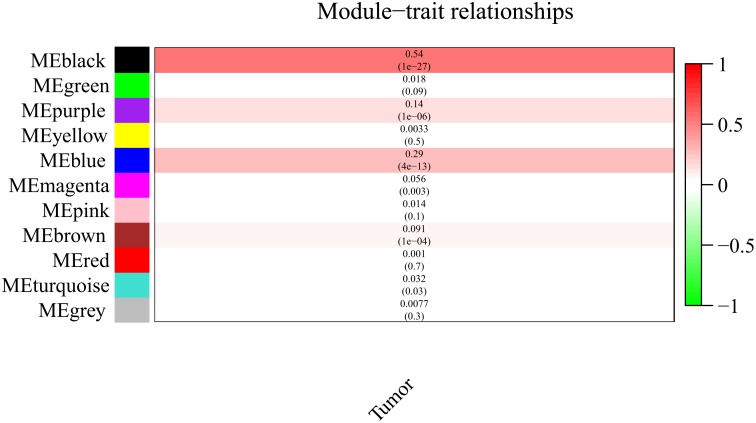
Heat map of correlation between eigengene modules and STS.

**FIGURE 6:**
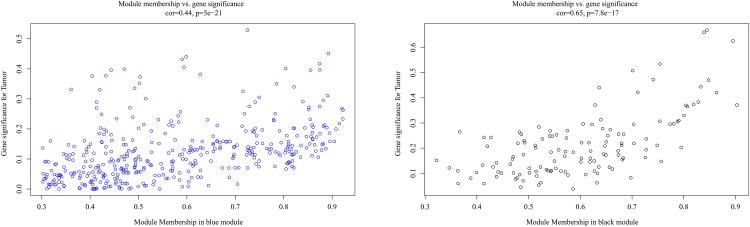
Scatter plot of eigengene modules in the blue and black modules.

It is important to identify the most significant modules related to STS. Both black and blue modules showed a significantly high correlation with sarcomas (Figure 5, 6). However, due to the lack of stability of the statistical data (Zsummary < 10), the black module was not further analyzed. Therefore, the blue module was defined as an important module of clinical significance and extracted for further analysis.

**FIGURE 7:**
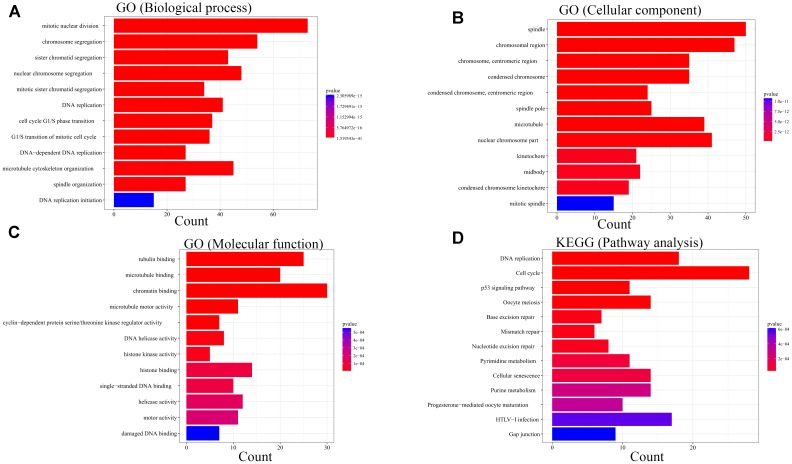
Bioinformatic analysis of genes in the blue module. GO analysis: **(A)** Biological process. **(B)** Cellular component. **(C)** Molecular function. KEGG analysis:**(D)** Pathway analysis.

For the sake of exploration of the biological relevance of the blue module, GO functional and KEGG pathway enrichment analyses were performed on 414 genes in the blue module. The biological processes of the genes in the blue module were found to associate with the cell cycle, such as mitotic nuclear division, chromosome segregation and sister chromatid segregation. In the KEGG pathway analysis, cell cycle associated signaling pathways such as DNA replication, cell cycle, p53 signaling pathway, oocyte meiosis, mismatch repair and metabolism associated pathways such as pyrimidine metabolism and purine metabolism were enriched (Figure 7).

### Identification of Sarcoma Hub Genes in the Blue Module

**FIGURE 8:**
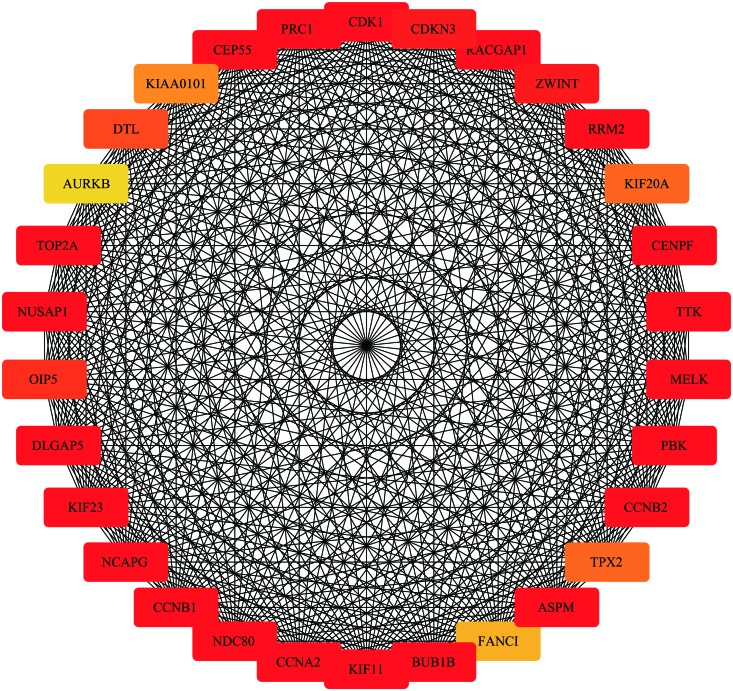
Protein-protein interaction network of the top 30 genes in the blue module (Node color: deeper colors indicates higher scores in the MCC analysis).

Highly connected hub genes within a module perform important roles in tumor biological processes. Therefore, the 20 genes with greatest module relevance in the blue module were selected as candidate hub genes for STS (Supplementary Data Sheet S1). In addition, a PPI network in the blue module was constructed in accordance with the STRING database (Figure 8). Twelve of the 20 candidate genes in the co-expression network were also identified as hub nodes of the PPI network. Finally, these 12 genes were considered “primary” hub genes associated with STS and therefore selected for additional analyses.

### Survival Analysis and Efficacy Evaluation

**FIGURE 9:**
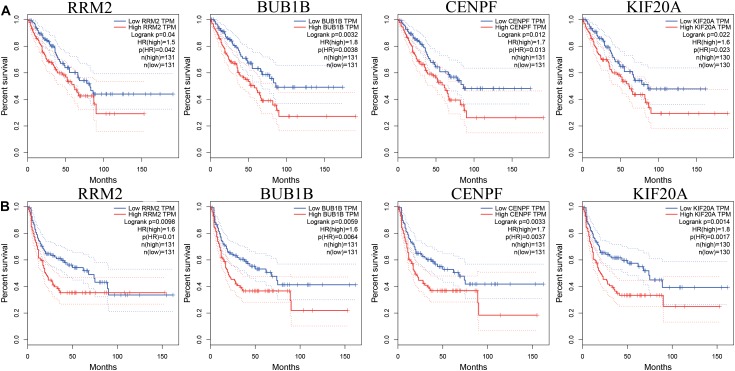
Survival analysis of association between RRM2, BUB1B, CENPF, and KIF20A expression levels and survival rates in STS based on TCGA microarray data. **(A)** Overall survival analysis. **(B)** Disease free survival.

**FIGURE 10:**
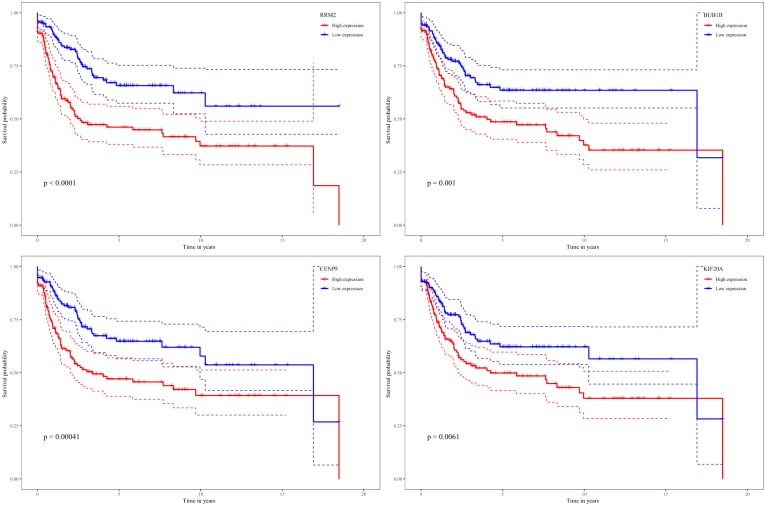
Survival analysis of association between RRM2, BUB1B, CENPF, and KIF20A expression levels and metastasis-free survival rates in STS based on GSE21050 microarray data.

**FIGURE 11:**
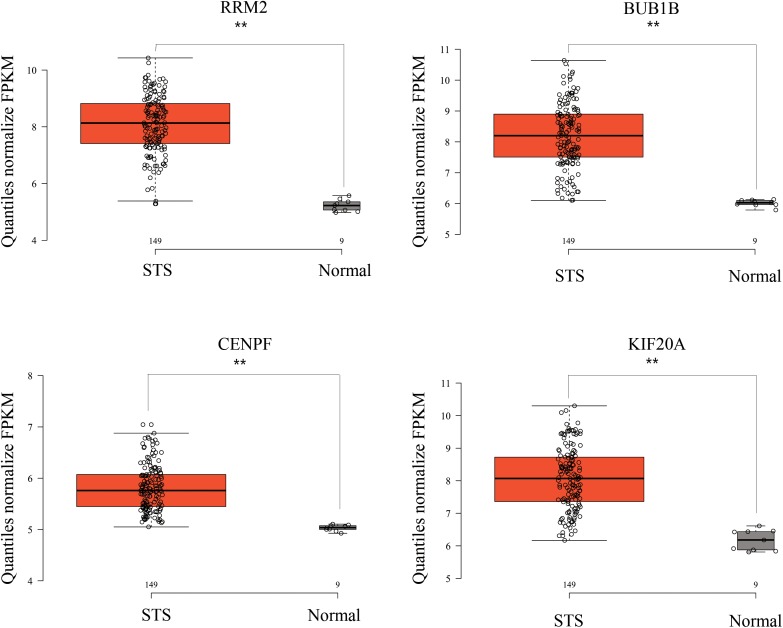
RRM2, BUB1B, CENPF, and KIF20A were strongly upregulated in STS tissues compared to normal fat tissue, based on GSE21122 microarray data. ^∗∗^*p* < 0.01.

While testing the TCGA dataset, four out of 12 hub genes demonstrated significant connectivity with overall and disease-free survival (Figure 9). When testing the GSE21050 dataset, these four hub genes showed significant correlation with metastasis free survival (Figure 10). Furthermore, they were significantly highly expressed in STS tissue compared to normal fat tissue (Figure 11).

### Gene Set Enrichment Analysis

**FIGURE 12:**
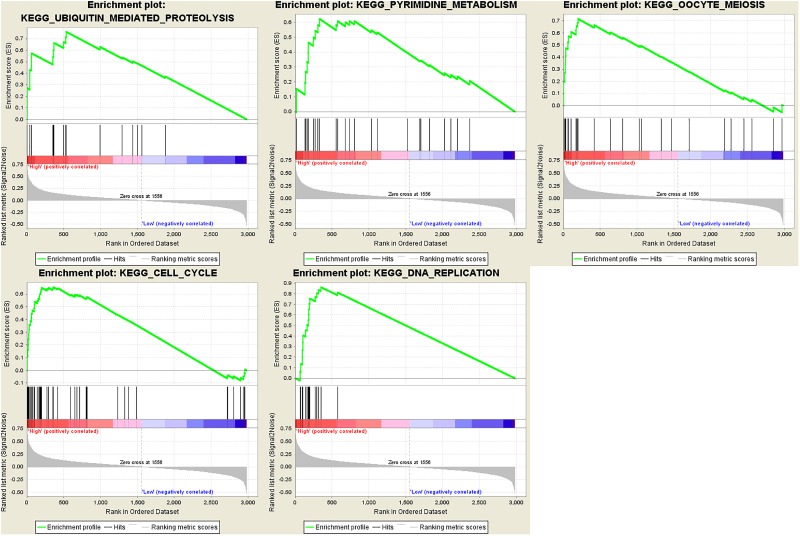
Gene set enrichment analysis (GSEA). Cell cycle and metabolism associated pathways were enriched.

In order to find out the potential function of both blue module and hub genes, GSEA was performed to identify KEGG pathways enriched in samples with higher level of ME of blue module. In GSEA analysis, five signaling pathways were significantly enriched, including ubiquitin mediated proteolysis (FDR = 0.01), pyrimidine metabolism (FDR = 0.03), oocyte meiosis (FDR = 0.02), cell cycle (FDR = 0.04) and DNA replication (FDR = 0.04) (Figure 12). Moreover, the last four pathways were consistent with the results of KEGG pathway analysis (Figure 7D).

## Discussion

Soft tissue sarcomas remain among the most challenging diseases for medical oncologists to treat. STSs are mesenchymal neoplasms that can arise from any site within the body, including extremities, the trunk, retroperitoneum, head, and neck. These are biologically heterogeneous diseases of which greater than 50 subtypes exist, varying by molecular, histological and clinical characteristics.

In this study, WGCNA was utilized to construct a co-expression network for identification of gene co-expression modules associated with STS. The blue module was positively identified and 20 hub genes selected from this module. In addition, as a result of the PPI network, 12 genes were identified as hub nodes of the co-expression module and PPI network, indicating that these 12 hub genes were closely related to STS and had important biological significance. Subsequent survival analysis established that four of the 12 hub genes (*RRM2, BUB1B, CENPF*, and *KIF20A*) were significantly associated with survival. We, therefore, focused on these four genes.

The ribonucleotide reductase regulatory subunit M2 (RRM2) is one of two subunits that constitute ribonucleotide reductase, the enzyme responsible for catalyzing the conversion of ribonucleotides into deoxyribonucleotides, and thus performing an important role in DNA synthesis. RRM2 is important in controlling cellular function in a number of human malignant tumors, including DNA repair, cell proliferation and senescence. Importantly, RRM2 functions as a driver in a variety of tumors, with *in vivo* and *in vitro* experiments confirming that knocking down expression using siRNA significantly inhibits tumor cell proliferation (Fang et al., 2016).

The BUB1 mitotic checkpoint serine/threonine kinase B (BUB1B) is a member of the spindle assembly checkpoint protein family, crucial for ensuring correct chromosome separation during cell division (Fu et al., 2016). BUB1B perfoms a role in the inhibition of APC expression, established as a tumor suppressor gene in most colorectal cancers. Accordingly, many reports have shown that upregulation of BUB1B is related to the recurrence and progression of bladder cancer (Yamamoto et al., 2007), gastric cancer (Ando et al., 2010), esophageal squamous cell carcinoma (Tanaka et al., 2008), breast cancer (Yuan et al., 2006), hepatocellular carcinoma (Liu et al., 2009) and others.

Centromere protein F (CENPF) is another important protein involved in chromosome segregation during mitosis. Upregulation of CENPF protein expression, especially through a gene amplification effect, suggests that high levels of CENPF protein may affect the occurrence of tumors, especially in the early stages of tumor development (Varis et al., 2006). Clinical research has demonstrated that high expression levels of CENPF results in poor prognosis in nasopharyngeal carcinoma (Cao et al., 2010), colorectal gastrointestinal stromal tumors (Chen et al., 2011), esophageal squamous cell carcinoma (Mi et al., 2013) and prostate cancer (Zhuo et al., 2015). It has also been shown to play an important role in driving hepatocellular carcinoma (Dai et al., 2013).

Kinesin family member 20A (KIF20A, also known as RAB6KIFL) belongs to the kinesin superfamily-6, located in the Golgi apparatus and contributes to intracellular organelle transport and cell division (Echard et al., 1998). Recently, it has been reported that KIF20A is associated with mitosis, cell adhesion, migration and proliferation. Furthermore, recent studies have demonstrated that KIF20A is involved in tumor progression and angiogenesis. High expression of KIF20A results poor prognosis in glioma patients (Duan et al., 2016; Saito et al., 2017), nasopharyngeal cancer (Liu et al., 2017), hepatocellular carcinoma (Shi et al., 2016), melanoma (Yamashita et al., 2012) and early-stage cervical squamous cell carcinoma (Zhang et al., 2016).

Regarding GSEA, it was found that cell cycle and metabolism associated pathways were significant enriched in samples with higher level of ME of blue module. This is consistent with the initial GO and KEGG analysis results of the blue module and are related to the physiological function of these four hub genes.

In summary, through WGCNA and other related analysis methods, we identified four genes (*RRM2, BUB1B, CENPF*, and *KIF20A*) related to the progression and prognosis of STS. These genes may play a role by regulating the cell cycle and metabolism associated signaling pathways.

## Author Contributions

ZZ and DS designed the study. ZZ and ZJ performed the data collection. ZJ and LW performed the data analysis. ZZ and MZ drafted the manuscript. All authors read and approved the final version of the manuscript.

## Conflict of Interest Statement

The authors declare that the research was conducted in the absence of any commercial or financial relationships that could be construed as a potential conflict of interest.

## Footnotes

1 https://genome-cancer.ucsc.edu/

2 http://www.string-db.org

3 http://gepia.cancer-pku.cn

## Supplementary Material

FIGURE S1 Data quality examination.

TABLE S1 Code for WGCNA.

DATA SHEET S1 Hub genes of blue module.

## References

[B1] Ando K. Kakeji Y. Kitao H. Iimori M. Zhao Y. Yoshida R. (2010). High expression of BUBR1 is one of the factors for inducing DNA aneuploidy and progression in gastric cancer. *Cancer Science.* 101 639–645. 10.1111/j.1349-7006.2009.01457.x 20132214 PMC11159402

[B2] Barretina J. Taylor B. S. Banerji S. Ramos A. H. Lagos-Quintana M. Decarolis P. L. (2010). Subtype-specific genomic alterations define new targets for soft-tissue sarcoma therapy. *Nat. Genet.* 42 715–721. 10.1038/ng.619 20601955 PMC2911503

[B3] Beck A. H. West R. B. van de Rijn M. (2010). Gene expression profiling for the investigation of soft tissue sarcoma pathogenesis and the identification of diagnostic, prognostic, and predictive biomarkers. *Virchows Archiv.* 456 141–151. 10.1007/s00428-009-0774-2 19412622 PMC4847139

[B4] Cao J. Y. Liu L. Chen S. P. Zhang X. Mi Y. J. Liu Z. G. (2010). Prognostic significance and therapeutic implications of centromere protein F expression in human nasopharyngeal carcinoma. *Mol. Cancer* 9 :237. 10.1186/1476-4598-9-237 20828406 PMC2944187

[B5] Casali P. G. Abecassis N. Bauer S. Biagini R. Bielack S. Bonvalot S. (2018). Soft tissue and visceral sarcomas: ESMO-EURACAN clinical practice guidelines for diagnosis, treatment and follow-up. *Annals. of Oncology.* 29 51–67. 10.1093/annonc/mdy096 29846498

[B6] Chen W. B. Cheng X. B. Ding W. Wang Y. J. Chen D. Wang J. H. (2011). Centromere protein F and survivin are associated with high risk and a poor prognosis in colorectal gastrointestinal stromal tumours. *J. Clin. Pathol.* 64 751–755. 10.1136/jcp.2011.089631 21613637

[B7] Chibon F. Lagarde P. Salas S. Perot G. Brouste V. Tirode F. (2010). Validated prediction of clinical outcome in sarcomas and multiple types of cancer on the basis of a gene expression signature related to genome complexity. *Nat. Med.* 16 781–787. 10.1038/nm.2174 20581836

[B8] Chin C. H. Chen S. H. Wu H. H. Ho C. W. Ko M. T. Lin C. Y. (2014). cytoHubba: identifying hub objects and sub-networks from complex interactome. *BMC Syst. Biol.* 8(Suppl. 4):S11. 10.1186/1752-0509-8-S4-S11 25521941 PMC4290687

[B9] Dai Y. Liu L. Zeng T. Zhu Y. H. Li J. Chen L. (2013). Characterization of the oncogenic function of centromere protein F in hepatocellular carcinoma. *Biochem. Biophys. Res. Commun.* 436 711–718. 10.1016/j.bbrc.2013.06.021 23791740

[B10] Duan J. Huang W. Shi H. (2016). Positive expression of KIF20A indicates poor prognosis of glioma patients. *Onco Targets Ther.* 9 6741–6749. 10.2147/OTT.S115974 27843327 PMC5098585

[B11] Echard A. Jollivet F. Martinez O. Lacapere J. J. Rousselet A. Janoueix-Lerosey I. (1998). Interaction of a Golgi-associated kinesin-like protein with Rab6. *Science* 279 580–585. 10.1126/science.279.5350.580 9438855

[B12] Fang Z. Lin A. Chen J. Zhang X. Liu H. Li H. (2016). CREB1 directly activates the transcription of ribonucleotide reductase small subunit M2 and promotes the aggressiveness of human colorectal cancer. *Oncotarget* 7 78055–78068. 10.18632/oncotarget.12938 27801665 PMC5363643

[B13] Fu X. Chen G. Cai Z. D. Wang C. Liu Z. Z. Lin Z. Y. (2016). Overexpression of BUB1B contributes to progression of prostate cancer and predicts poor outcome in patients with prostate cancer. *Onco Targets Ther.* 9 2211–2220. 10.2147/OTT.S101994 27143916 PMC4844448

[B14] Greither T. Wedler A. Rot S. Kessler J. Kehlen A. Holzhausen H. J. (2017). CMG2 expression is an independent prognostic factor for soft tissue sarcoma patients. *International. Journal. of Molecular. Sciences.* 18 :E2648. 10.3390/ijms18122648 29215551 PMC5751250

[B15] Langfelder P. Horvath S. (2008). WGCNA: an R package for weighted correlation network analysis. *BMC Bioinformatics* 9:559. 10.1186/1471-2105-9-559 19114008 PMC2631488

[B16] Langfelder P. Luo R. Oldham M. C. Horvath S. (2011). Is my network module preserved and reproducible? *PLoS Comput. Biol.* 7:e1001057. 10.1371/journal.pcbi.1001057 21283776 PMC3024255

[B17] Li J. C. A. (2003). Modeling survival data: extending the Cox model. *Sociological. Methods & Research.* 32 117–120. 10.1177/0049124103031004005

[B18] Linch M. Miah A. B. Thway K. Judson I. R. Benson C. (2014). Systemic treatment of soft-tissue sarcoma-gold standard and novel therapies. *Nat. Rev. Clin. Oncol.* 11 187–202. 10.1038/nrclinonc.2014.26 24642677

[B19] Liu A. W. Cai J. Zhao X. L. Xu A. M. Fu H. Q. Nian H. (2009). The clinicopathological significance of BUBR1 overexpression in hepatocellular carcinoma. *J. Clin. Pathol.* 62 1003–1008. 10.1136/jcp.2009.066944 19861558

[B20] Liu S. L. Lin H. X. Qiu F. Zhang W. J. Niu C. H. Wen W. (2017). Overexpression of kinesin family member 20A correlates with disease progression and poor prognosis in human nasopharyngeal cancer: a retrospective analysis of 105 patients. *PLoS One* 12:e0169280. 10.1371/journal.pone.0169280 28081138 PMC5230771

[B21] Mi Y. J. Gao J. Xie J. D. Cao J. Y. Cui S. X. Gao H. J. (2013). Prognostic relevance and therapeutic implications of centromere protein F expression in patients with esophageal squamous cell carcinoma. *Dis. Esophagus* 26 636–643. 10.1111/dote.12002 23163484

[B22] Nakazawa M. S. Eisinger-Mathason T. S. Sadri N. Ochocki J. D. Gade T. P. Amin R. K. (2016). Epigenetic re-expression of HIF-2alpha suppresses soft tissue sarcoma growth. *Nat. Commun.* 7 :10539. 10.1038/ncomms10539 26837714 PMC4742834

[B23] Saito K. Ohta S. Kawakami Y. Yoshida K. Toda M. (2017). Functional analysis of KIF20A, a potential immunotherapeutic target for glioma. *J. Neurooncol.* 132 63–74. 10.1007/s11060-016-2360-1 28070829

[B24] Shi C. Huang D. L. Lu N. H. Chen D. Zhang M. H. Yan Y. H. (2016). Aberrantly activated Gli2-KIF20A axis is crucial for growth of hepatocellular carcinoma and predicts poor prognosis. *Oncotarget* 7 26206–26219. 10.18632/oncotarget.8441 27036048 PMC5041975

[B25] Smolle M. A. Leithner A. Posch F. Szkandera J. Liegl-Atzwanger B. Pichler M. (2017). MicroRNAs in different histologies of soft tissue sarcoma: a comprehensive review. *International. Journal. of Molecular. Sciences.* 18 :E1960. 10.3390/ijms18091960 28895916 PMC5618609

[B26] Takahashi A. Nakayama R. Ishibashi N. Doi A. Ichinohe R. Ikuyo Y. (2014). Analysis of gene expression profiles of soft tissue sarcoma using a combination of knowledge-based filtering with integration of multiple statistics. *PLoS One* 9:e106801. 10.1371/journal.pone.0106801 25188299 PMC4154757

[B27] Tanaka K. Mohri Y. Ohi M. Yokoe T. Koike Y. Morimoto Y. (2008). Mitotic checkpoint genes, hsMAD2 and BubR1, in oesophageal squamous cancer cells and their association with 5-fluorouracil and cisplatin-based radiochemotherapy. *Clinical. Oncology.* 20 639–646. 10.1016/j.clon.2008.06.010 18691855

[B28] Varis A. Salmela A. L. Kallio M. J. (2006). Cenp-F (mitosin) is more than a mitotic marker. *Chromosoma* 115 288–295. 10.1007/s00412-005-0046-0 16565862

[B29] Yamamoto Y. Matsuyama H. Chochi Y. Okuda M. Kawauchi S. Inoue R. (2007). Overexpression of BUBR1 is associated with chromosomal instability in bladder cancer. *Cancer Genetics. and Cytogenetics.* 174 42–47. 10.1016/j.cancergencyto.2006.11.012 17350465

[B30] Yamashita J. Fukushima S. Jinnin M. Honda N. Makino K. Sakai K. (2012). Kinesin family member 20A is a novel melanoma-associated antigen. *Acta Dermato-venereologica.* 92 593–597. 10.2340/00015555-1416 22854760

[B31] Yang J. L. Das Gupta R. Goldstein D. Crowe P. J. (2017). Significance of phosphorylated epidermal growth factor receptor and its signal transducers in human soft tissue sarcoma. *International. Journal. of Molecular. Sciences.* 18 :E1159. 10.3390/ijms18061159 28556791 PMC5485983

[B32] Yu G. C. Wang L. G. Han Y. Y. He Q. Y. (2012). clusterProfiler: an r package for comparing biological themes among gene clusters. *Omics-a Journal. of Integrative. Biology.* 16 284–287. 10.1089/omi.2011.0118 22455463 PMC3339379

[B33] Yuan B. B. Xu Y. Woo J. H. Wang Y. Y. Bae Y. K. Yoon D. S. (2006). Increased expression of mitotic checkpoint genes in breast cancer cells with chromosomal instability. *Clinical. Cancer Research.* 12 405–410. 10.1158/1078-0432.Ccr-05-0903 16428479

[B34] Zhang W. J. He W. L. Shi Y. J. Gu H. F. Li M. Liu Z. M. (2016). High expression of KIF20A is associated with poor overall survival and tumor progression in early-stage cervical squamous cell carcinoma. *PLoS One* 11:e0167449. 10.1371/journal.pone.0167449 27941992 PMC5152822

[B35] Zhuo Y. J. Xi M. Wan Y. P. Hua W. Liu Y. L. Wan S. (2015). Enhanced expression of centromere protein F predicts clinical progression and prognosis in patients with prostate cancer. *International. Journal. of Molecular. Medicine.* 35 966–972. 10.3892/ijmm.2015.2086 25647485

