# Supplementary figures and images for: Co-expression Network Analysis Identifies Four Hub Genes Associated With Prognosis in Soft Tissue Sarcoma
Source: Front Genet. 2019 Feb 4;10:37. doi: 10.3389/fgene.2019.00037 (PMC6369179; doi:10.3389/fgene.2019.00037)

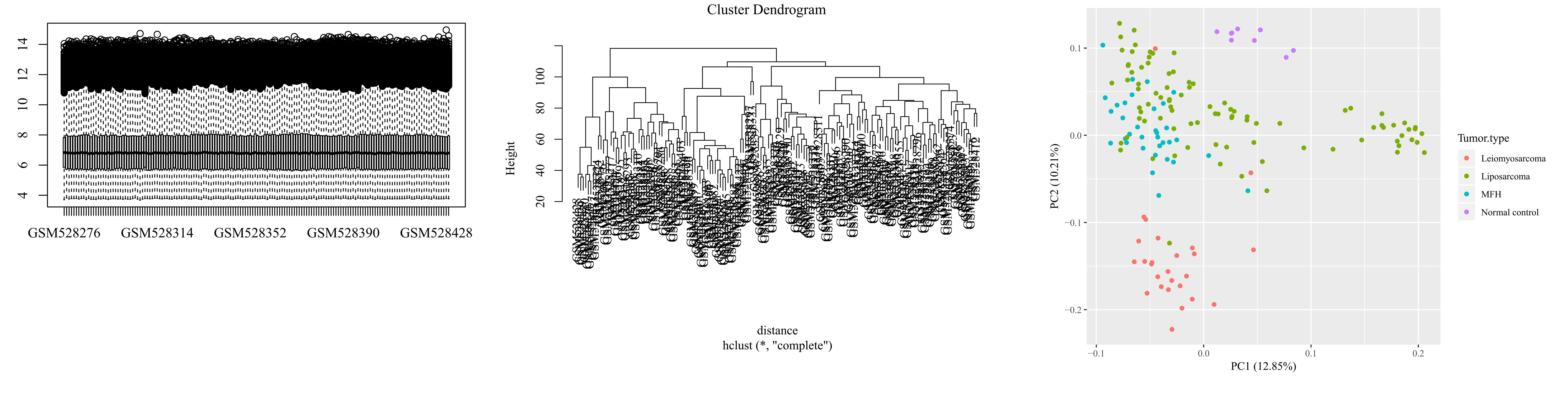

Supplement: FIGURE S1 — Data quality examination. [file Image_1.TIFF]
