# Supplementary material for: Co-expression Network Analysis Identifies Four Hub Genes Associated With Prognosis in Soft Tissue Sarcoma
Source: Front Genet. 2019 Feb 4;10:37. doi: 10.3389/fgene.2019.00037 (PMC6369179; doi:10.3389/fgene.2019.00037)
Supplement: TABLE S1 — Code for WGCNA. [file Table_1.DOCX]

###Install the necessary packages

if (!requireNamespace("BiocManager", quietly = TRUE))

install.packages("BiocManager")

BiocManager::install("limma", version = "3.8")

BiocManager::install("WGCNA", version = "3.8")

BiocManager::install("clusterProfiler", version = "3.8")

install.packages("stringr")

install.packages("ggplot2")

install.packages("ggfortify")

install.packages("xlsx")

install.packages("survival")

###load packages

library(WGCNA)

allowWGCNAThreads()

options(stringsAsFactors = FALSE)

rm(list = ls())

###GSE21122 MINiML file was downloaded from GEO, expression files were extracted and merged

##set work path, which contains the merged expression matrix of GSE21122

setwd("C:\\Users\\Zhu\\Desktop\\STS_WGCNA")

datExpr <- read.table("GSE21122_exp_processed.txt",header = T,row.names = 1,sep = "\t")

###data assesment (batch effect?)

library(ggfortify)

library(xlsx)

datTraits <- read.xlsx("datTraits.xlsx",sheetIndex = 1) ###samples information

boxplot(datExpr)

distance <- dist(t(datExpr),method="euclidean")

clusters <- hclust(distance)

plot(clusters)

##PCA plot

pca.rma<-prcomp(t(datExpr))

autoplot(pca.rma, data = datTraits, colour = 'Tumor.type')

##No significant batch effect was found

###############Data Processing####################

dim(datExpr) ##genes=13435 samples=158

datExpr <- t(datExpr)

rownames(datTraits) <- datTraits$Accession

datTraits <- datTraits[,-1]

head(datTraits)

all(rownames(datExpr)==rownames(datTraits)) ##samples names matcthed

gsg <- goodSamplesGenes(datExpr)

gsg$allOK ##True. All samples and genes meet quality standards.

##detectting outliers

A = adjacency(t(datExpr), type = "distance")

# this calculates the whole network connectivity

k = as.numeric(apply(A, 2, sum)) - 1

# standardized connectivity

Z.k = scale(k)

# Designate samples as outlying if their Z.k value is below the threshold

thresholdZ.k = -0.6

# the color vector indicates outlyingness (red)

outlierColor = ifelse(Z.k < thresholdZ.k, "red", "black")

# calculate the cluster tree using flahsClust or hclust

sampleTree = hclust(as.dist(1 - A), method = "average")

# Convert traits to a color representation: where red indicates high

# Plot the sample dendrogram and the colors underneath.

plotDendroAndColors(sampleTree, groupLabels = "Outliers", colors = outlierColor,

main = "Sample dendrogram and trait heatmap")

outliers <- which(Z.k< - 0.6)

rownames(datExpr)[outliers]

##outliers: "GSM528297" "GSM528333"

datExpr <- datExpr[-outliers,]

datTraits <- datTraits[-outliers,]

#### the top 3000 most variant genes were used for the following analysis

datExpr <- datExpr[,order(apply(datExpr,2,mad),decreasing = T)[1:3000]]

save(datExpr,datTraits,file = "DatExpr&Traits.RData")

setwd("C:\\Users\\Zhu\\Desktop\\STS_WGCNA")

load("DatExpr&Traits.RData")

###Pick soft power

powers = c(c(1:10), seq(from = 12, to=20, by=2))

sft = pickSoftThreshold(datExpr, powerVector = powers,verbose = 3)

# Plot the results:

par(mfrow = c(1, 2))

# SFT index as a function of different powers

plot(sft$fitIndices[, 1], -sign(sft$fitIndices[, 3]) * sft$fitIndices[, 2],

xlab = "Soft Threshold (power)", ylab = "SFT, signed R^2", type = "n", main = paste("Scale independence"))

text(sft$fitIndices[, 1], -sign(sft$fitIndices[, 3]) * sft$fitIndices[, 2],

labels = powers, col = "red")

# this line corresponds to using an R^2 cut-off of h

abline(h = 0.9, col = "red")

# Mean connectivity as a function of different powers

plot(sft$fitIndices[, 1], sft$fitIndices[, 5], type = "n", xlab = "Soft Threshold (power)",

ylab = "Mean Connectivity", main = paste("Mean connectivity"))

text(sft$fitIndices[, 1], sft$fitIndices[, 5], labels = powers, col = "red")

text(sft$fitIndices[,1], sft$fitIndices[,5], labels=powers,col="red")

##building the co-expression network

power = 6 ###pick the soft power

net = blockwiseModules(

datExpr,

power = power,

maxBlockSize = 6000,

TOMType = "unsigned", minModuleSize = 30,

reassignThreshold = 0, mergeCutHeight = 0.25,

numericLabels = TRUE, pamRespectsDendro = FALSE,

verbose = 3

)

table(net$colors)

# Convert labels to colors for plotting

mergedColors = labels2colors(net$colors)

table(mergedColors)

# Plot the dendrogram and the module colors underneath

plotDendroAndColors(net$dendrograms[[1]], mergedColors[net$blockGenes[[1]]],

"Module colors",

dendroLabels = FALSE, hang = 0.03,

addGuide = TRUE, guideHang = 0.05)

save(MEs,net,mergedColors,file = "Co-expression_network.RData")

###module-trait correlation using linear regression method

nGenes = ncol(datExpr)

nSamples = nrow(datExpr)

moduleColors <- labels2colors(net$colors)

MEs0 <- moduleEigengenes(datExpr,moduleColors)$eigengenes

MEs <- orderMEs(MEs0)

design=model.matrix(~0+ datTraits$Tumor.Control)

colnames(design)=levels(factor(datTraits$Tumor.Control))

##calculate correlation between traits and MEs using line regression method

ModuleTraitLM <- matrix(data = NA,nrow = ncol(MEs),ncol = 1,dimnames = list(colnames(MEs),colnames(design)[2]))

ModuleTraitPvalue <- matrix(data = NA,nrow = ncol(MEs),ncol = 1,dimnames = list(colnames(MEs),colnames(design)[2]))

for(n in 1:ncol(MEs)){

Lm_model <- lm(MEs[,n]~design[,2])

Lm_summary <- summary(Lm_model)

ModuleTraitLM[n,1] <- Lm_summary$r.squared

f <- Lm_summary$fstatistic

ModuleTraitPvalue[n,1] <- pf(f[1], f[2], f[3], lower=FALSE)

}

textMatrix = paste(signif(ModuleTraitLM, 2), "\n(",

signif(ModuleTraitPvalue, 1), ")", sep = "")

dim(textMatrix) = dim(ModuleTraitLM)

par(mar = c(6, 8.5, 3, 3));

# Display the correlation values within a heatmap plot

labeledHeatmap(Matrix = ModuleTraitLM,

xLabels = "Tumor",

yLabels = names(MEs),

ySymbols = names(MEs),

colorLabels = FALSE,

colors = greenWhiteRed(50),

textMatrix = textMatrix,

setStdMargins = FALSE,

cex.text = 0.5,

zlim = c(-1,1),

main = paste("Module-trait relationships"))

###Module membership and gene significance

##Gene membership

modNames = substring(names(MEs), 3)

geneModuleMembership = as.data.frame(cor(datExpr, MEs, use = "p"))

MMPvalue = as.data.frame(corPvalueStudent(as.matrix(geneModuleMembership), nSamples))

names(geneModuleMembership) = paste("MM", modNames, sep="")

names(MMPvalue) = paste("p.MM", modNames, sep="")

##Gene traits relashionship using line regression model

Tumor <- as.data.frame(design[,2])

names(Tumor) <- "Tumor"

geneTraitSignificance <- matrix(data = NA,nrow = ncol(datExpr),ncol = 1,dimnames = list(colnames(datExpr),"Tumor"))

GSPvalue <- matrix(data = NA,nrow = ncol(datExpr),ncol = 1,dimnames = list(colnames(datExpr),"Tumor"))

for( n in 1:ncol(datExpr)){

Lm_model <- lm(datExpr[,n]~design[,2])

Lm_summary <- summary(Lm_model)

geneTraitSignificance[n,1] <- Lm_summary$r.squared

f <- Lm_summary$fstatistic

GSPvalue[n,1] <- pf(f[1], f[2], f[3], lower=FALSE)

}

names(geneTraitSignificance) = paste("GS.", names(Tumor), sep="")

names(GSPvalue) = paste("p.GS.", names(Tumor), sep="")

module = "blue"

#module = "black"

column = match(module, modNames)

moduleGenes = mergedColors==module

sizeGrWindow(7, 7)

par(mfrow = c(1,1))

verboseScatterplot(abs(geneModuleMembership[moduleGenes, column]),

abs(geneTraitSignificance[moduleGenes, 1]),

xlab = paste("Module Membership in", module, "module"),

ylab = "Gene significance for Tumor",

main = paste("Module membership vs. gene significance\n"),

cex.main = 1.2, cex.lab = 1.2, cex.axis = 1.2, col = module)

###Module preservation test

###FPKM expression data of STS was downloaded from TCGA-SARC projiec

###Then TPM normalized

Test <- read.table("TCGA_TPM.txt",header = T,row.names = 1,sep = "\t")

dim(Test) ## genes=34598 samples=265

Test <- t(Test)

gsg_test <- goodSamplesGenes(Test)

Test <- Test[gsg_test$goodSamples,gsg_test$goodGenes]

common_genes_index <- which(colnames(datExpr) %in% colnames(Test)) ##Total 2704 genes in common

datExpr <- datExpr[,common_genes_index] ##Retain only common genes

mergedColors <- mergedColors[common_genes_index] ##Retain the module color of common genes

Test <- Test[,colnames(datExpr)]

multiExpr <- list(Training=list(data=datExpr),Test=list(data=Test))

multiColor <- list(Training=mergedColors)

mp=modulePreservation(multiData = multiExpr,multiColor = multiColor,referenceNetworks = 1,nPermutations = 200,randomSeed = 1,quickCor = 0,verbose = 3)

save(mp, file = "modulePreservation.RData")

#load(file = "modulePreservation.RData")

##查看数据

ref = 1

test = 2

statsObs = cbind(mp$quality$observed[[ref]][[test]][, -1], mp$preservation$observed[[ref]][[test]][, -1])

statsZ = cbind(mp$quality$Z[[ref]][[test]][, -1], mp$preservation$Z[[ref]][[test]][, -1])

# Compare preservation to quality:

print( cbind(statsObs[, c("medianRank.pres", "medianRank.qual")],

signif(statsZ[, c("Zsummary.pres", "Zsummary.qual")], 2)) )

# Module labels and module sizes are also contained in the results

modColors = rownames(mp$preservation$observed[[ref]][[test]])

moduleSizes = mp$preservation$Z[[ref]][[test]][, 1];

# leave grey and gold modules out

plotMods = !(modColors %in% c("grey", "gold"));

# Text labels for points

text = modColors[plotMods];

# Auxiliary convenience variable

plotData = cbind(mp$preservation$observed[[ref]][[test]][, 2], mp$preservation$Z[[ref]][[test]][, 2])

# Main titles for the plot

mains = c("Preservation Median rank", "Preservation Zsummary");

# Start the plot

sizeGrWindow(10, 5);

#pdf(fi="Plots/BxHLiverFemaleOnly-modulePreservation-Zsummary-medianRank.pdf", wi=10, h=5)

par(mfrow = c(1,2))

par(mar = c(4.5,4.5,2.5,1))

for (p in 1:2)

{

min = min(plotData[, p], na.rm = TRUE);

max = max(plotData[, p], na.rm = TRUE);

# Adjust ploting ranges appropriately

if (min > -max/10) min = -max/10

ylim = c(min - 0.1 * (max-min), max + 0.1 * (max-min))

plot(moduleSizes[plotMods], plotData[plotMods, p], col = 1, bg = modColors[plotMods], pch = 21,

main = mains[p],

cex = 2.4,

ylab = mains[p], xlab = "Module size", log = "x",

ylim = ylim,

xlim = c(10, 2000), cex.lab = 1.2, cex.axis = 1.2, cex.main =1.4)

labelPoints(moduleSizes[plotMods], plotData[plotMods, p], text, cex = 1, offs = 0.08);

# For Zsummary, add threshold lines

if (p==2)

{

abline(h=0)

abline(h=2, col = "blue", lty = 2)

abline(h=10, col = "darkgreen", lty = 2)

}

}

# If plotting into a file, close it

#dev.off();

##reload data

rm(list = ls())

load("DatExpr&Traits.RData")

load("Co-expression_network.RData")

##Extract genes in blue module

# Select module

module = "blue"

# Select module probes

probes = colnames(datExpr)

inModule = (mergedColors==module)

modProbes = probes[inModule]

write.table(modProbes,"module_blue.txt",row.names = F,col.names = F,quote = F,sep = "\t")

##Hub genes in blue module

datKME=signedKME(datExpr, MEs, outputColumnName="MM.") #datKME=genemodulemembership

hubgenes <- rownames(datKME[order(abs(datKME$MM.blue),decreasing = T)[1:20],])

write.csv(hubgenes,"blue_hubgenes.csv")

# Recalculate topological overlap

TOM = TOMsimilarityFromExpr(datExpr, power = 6)

# Select the corresponding Topological Overlap

modTOM = TOM[inModule, inModule]

dimnames(modTOM) = list(modProbes, modProbes)

## The relationship between the nodes was exported to cytoscape

cyt = exportNetworkToCytoscape(

modTOM,

edgeFile = paste("CytoscapeInput-edges-", paste(module, collapse="-"), ".txt", sep=""),

nodeFile = paste("CytoscapeInput-nodes-", paste(module, collapse="-"), ".txt", sep=""),

weighted = TRUE,

threshold = 0.02,

nodeNames = modProbes,

nodeAttr = mergedColors[inModule]

)

##Function annotation

library(clusterProfiler)

library(org.Hs.eg.db)

rt <- read.table("module_blue.txt",colClasses = "character")

rt1 <- rt[,1]

eg <- bitr(rt1,fromType = "SYMBOL",toType = "ENTREZID",org.Hs.eg.db)

gene <- eg[,2]

ego_cc <- enrichGO(gene,org.Hs.eg.db,ont = "CC",pvalueCutoff = 0.05) #cellular compon

ego_bp <- enrichGO(gene,org.Hs.eg.db,ont = "BP",pvalueCutoff = 0.05) #biological process

ego_mf <- enrichGO(gene,org.Hs.eg.db,ont = "MF",pvalueCutoff = 0.05) #molecular funtion

kk <- enrichKEGG(gene,pvalueCutoff = 0.05,organism = "hsa") #kegg

#visualize

dotplot(ego_cc)

barplot(ego_cc,showCategory = 12)

dotplot(ego_bp)

barplot(ego_bp,showCategory = 12)

dotplot(ego_mf)

barplot(ego_mf,showCategory = 12)

barplot(kk,showCategory = 24)

##survival test in GSE21050

library(survival)

Expr_21050 <- read.table("Expr_21050.txt",header = T,row.names = 1,sep = "\t")

Traits_21050 <- read.table("Traits_21050.txt",header = T,row.names = 1,sep = "\t") ##310 samples;Metastasis status and time

head(Traits_21050)

colnames(Traits_21050) <- c("fustat","futime")

all(colnames(Expr_21050)==rownames(Traits_21050))

genename <- "KIF20A" ## sames for BUB1B, CENPF, KIF20A

a=Expr_21050[genename,]<median(as.numeric(Expr_21050[genename,])) ##group by hub gene expression

a <- as.character(a)

diff=survdiff(Surv(futime, fustat) ~a,data = Traits_21050)

pValue=1-pchisq(diff$chisq,df=1)

pValue=round(pValue,5)

fit <- survfit(Surv(futime, fustat) ~ a, data = Traits_21050)

plot(fit, lty = 2:3,col=c("red","blue"),xlab="time (month)",ylab="surival rate",

main=paste(genename,"(p=", pValue ,")",sep=" "))

legend("topright", c(paste(genename,"high expression",sep=" "), paste(genename,"low expression",sep=" ")), lty = 2:3, col=c("red","blue"))
